# Supplementary material for: A novel approach to porcine abnormal sounds recognition based on improved Multi-SVDD
Source: PLoS One. 2025 Sep 29;20(9):e0332996. doi: 10.1371/journal.pone.0332996 (PMC12478944; doi:10.1371/journal.pone.0332996)
Supplement: Attachment — Submitted filename: Response to Reviewers.docx [file pone.0332996.s002.docx]

Dear Reviewers:

Thank you for your letter and the reviewers’ comments on our manuscript entitled " A novel approach to porcine abnormal sounds recognition based on improved Multi-SVDD" (ID PONE-D-25-35613). Those comments are very helpful for revising and improving our paper, as well as the important guiding significance to other research. We have studied the comments carefully and made corrections which we hope meet with approval. The main corrections are in the manuscript and the responds to the reviewers’ comments are as follows (the replies are highlighted in blue ).

Replies to the journal requirements:

1. Please ensure that your manuscript meets PLOS ONE's style requirements, including those for file naming. The PLOS ONE style templates can be found at

[https://journals.plos.org/plosone/s/file?id=wjVg/PLOSOne_formatting_sample_main_body.pdf](https://journals.plos.org/plosone/s/file?id=wjVg/PLOSOne_formatting_sample_main_body.pdf) and

[https://journals.plos.org/plosone/s/file?id=ba62/PLOSOne_formatting_sample_title_authors_affiliations.pdf](https://track.editorialmanager.com/CL0/https://journals.plos.org/plosone/s/file?id=ba62/PLOSOne_formatting_sample_title_authors_affiliations.pdf/1/010f01983d0eecb8-6108bf3a-343a-4df6-bb89-4f0e85046dc6-000000/EL3MKKxc6I9LJQX-2qfpjQgXdsVsjVLuNNi9M26klWU=221)

Response: Thank you for pointing this out. We have adjusted the format of the manuscript according to the PLOS ONE style templates.

2. Please note that PLOS One has specific guidelines on code sharing for submissions in which author-generated code underpins the findings in the manuscript. In these cases, we expect all author-generated code to be made available without restrictions upon publication of the work. Please review our guidelines at [https://journals.plos.org/plosone/s/materials-and-software-sharing#loc-sharing-code](https://journals.plos.org/plosone/s/materials-and-software-sharing#loc-sharing-code) and ensure that your code is shared in a way that follows best practice and facilitates reproducibility and reuse.

Response: I'm very sorry, but due to legal and specific regulatory requirements, I am unable to share the code. Should you require access to the code, please feel free to contact the corresponding author.

3. Thank you for stating the following financial disclosure:
   “This research work was funded by the Fundamental Research Program of Shanxi Province (Grant No. 202303021222303), Scientific and Technological Innovation Programs of Higher Education Institutions in Shanxi (Grant No. 2023L362) and Taiyuan Institute of Technology Science Research Initial Funding (Grant No. 2022LJ021).”

Please state what role the funders took in the study. If the funders had no role, please state: "The funders had no role in study design, data collection and analysis, decision to publish, or preparation of the manuscript." If this statement is not correct you must amend it as needed. Please include this amended Role of Funder statement in your cover letter; we will change the online submission form on your behalf.

Response: Thanks for your suggestions. This sentence has been added in lines 458–459 on page 20.

4. We note that the grant information you provided in the ‘Funding Information’ and ‘Financial Disclosure’ sections do not match.
   When you resubmit, please ensure that you provide the correct grant numbers for the awards you received for your study in the ‘Funding Information’ section.

Response: Thank you for pointing this out. We have carefully checked the ‘Funding Information’ section and confirmed that the grant numbers are correct.

5. Please include captions for your Supporting Information files at the end of your manuscript, and update any in-text citations to match accordingly. Please see our Supporting Information guidelines for more information: [http://journals.plos.org/plosone/s/supporting-information](http://journals.plos.org/plosone/s/supporting-information).

Response: Thank you for pointing this out. We have added Supporting Information at the end of the manuscript.

6. We are unable to open your Supporting Information file “Figures.rar”. Please kindly revise as necessary and re-upload.

Response: Thank you for pointing this out. We have renamed file “Figures.rar” to "S1-S8 Figs.zip" and re-uploaded it.

7. If the reviewer comments include a recommendation to cite specific previously published works, please review and evaluate these publications to determine whether they are relevant and should be cited. There is no requirement to cite these works unless the editor has indicated otherwise.

Response: Thank you for pointing this out.

Replies to the first reviewer’s comments:

1.The current manuscript is primarily driven by performance improvement in sound recognition. It is recommended that the Introduction clearly articulate the key challenges in animal sound recognition, such as difficulties in data annotation, ambiguity in abnormal categories, and complex background noise. The necessity of the proposed method should be justified from a scientific problem-solving perspective.

Response: We appreciate the reviewer’s valuable suggestions. The key challenges in animal sound recognition have been incorporated into the introduction, specifically detailed in lines 51–56 on pages 2 and 3.

2.The authors should discuss the potential generalizability of the proposed method to other animal species or even to general unstructured acoustic environments. Emphasizing this extension would enhance the impact and applicability of the work.

Response: Thanks for the reviewer’s kind suggestion. We have discussed the advantages of the proposed method in this paper, as well as the domains in which it can be extended and applied. These details have been added in lines 71-73 on page 3 and lines 410-414 on page 19.

3.The mathematical derivation of standard SVDD occupies a significant portion of the manuscript. It is suggested to streamline this part by moving the well-known steps to an appendix or omitting them, and instead highlighting the proposed density-weighted modification and its core equations.

Response: We sincerely appreciate the reviewer’s thoughtful suggestion. The mathematical derivation of the standard SVDD has been omitted, and the key steps of the improved Multi-SVDD have been emphasized. These revisions are detailed in lines 246–283 on pages 11 to 13.

4.Parts of Figures 1 and 6–7 appear blurry and lack detailed annotations. It is recommended to improve the visual clarity and label axes explicitly (e.g., time, frequency, MFCC coefficient index) to enhance interpretability.

Response: Thanks for the reviewer’s kind suggestion. In the spectrograms of Fig 1, color is used to represent amplitude. Specifically, brighter colors indicate higher amplitudes, while darker colors correspond to lower amplitudes. The detailed annotations have been added in lines 90-91 on page 4. The visual clarity of Fig 6 and Fig 7 has been improved.

5.The titles of the tables can be refined for clarity and conciseness. For instance, the title of Table 4 may be revised to: “Comparison of different classification methods on porcine abnormal sound recognition.”

Response: Thanks for the reviewer’s suggestions. The titles of Table 1 to 4 have been refined to more clearly and accurately reflect the content of the tables.

6.The conclusion section should go beyond summarizing results. The authors are encouraged to discuss the practical feasibility of deploying the proposed method at scale, potential directions for improvement (e.g., joint localization and recognition, real-time implementation), and remaining open challenges (e.g., multi-class detection, data scarcity, and inference efficiency).

Response: Thank you for pointing this out. The drawback of the present paper is that it may cause recognition errors when collecting mixtures of different types of porcine sounds. Additionally, when porcine abnormal sounds are detected, the specific location of the abnormal pigs cannot be determined. Our future work will focus on extracting effective sounds from mishmashed sounds and fusing the abnormal sounds recognition and localization. In future work, blind source separation methods will be investigated to separate mixed sounds, and the accurate localization of sound sources following porcine abnormal sound recognition will also be explored. These details have been added in lines 414-421 on pages 19 to 20.

7.While this work is focused on porcine sound recognition, the proposed formulation is applicable to broader time-series classification and detection tasks. The authors may consider citing related works such as “Fault detection for point machines: A review, challenges, and perspectives” and “Quantification of abnormal characteristics and flow-patterns identification in pumped storage system” to highlight this potential.

Response: Thanks for the reviewer’s kind suggestion. SVDD is a widely utilized One-Class Classification (OCC) method designed to classify positive cases without well-defined negative cases. SVDD is widely applied in fields such as fault detection and anomaly identification. It is presented in the introduction in lines 58-60 on page 3. The references are cited in this section.

8.The open-source link provided in the manuscript is currently inaccessible, and the dataset cannot be downloaded. The authors are advised to update the link or host the dataset on a stable and publicly accessible repository to ensure reproducibility.

Response: Thank you for pointing this out. We have updated the dataset again. It can be downloaded normally now.

Replies to the second reviewer’s comments:

1. The Introduction only briefly mentions deep neural networks. The authors should explain why their approach outperforms deep-learning alternatives to clarify the research motivation.

Response: Thanks for the reviewer’s kind suggestion. The acoustic environment in a real pigpen is significantly more complex. In addition to porcine abnormal sounds, there are many other kinds of sounds in the pen. There is a serious imbalance in the number of these kinds of sounds. Consequently, annotating these additional sound types is both time-consuming and challenging, making the accurate detection of porcine abnormal sounds from the entire set of collected sounds particularly difficult. Moreover, recognizing the collected sounds by classification algorithm may classify the other kinds of sounds as porcine abnormal sounds. When training data are incorrectly tagged, the recognition accuracy will be adversely influenced. These problems all limit the accuracy and stability of classification algorithms, including deep neural network, in identifying abnormal sounds of pigs. The method proposed in this paper can recognize porcine cough and scream from all the collected sounds and improve the accuracy and stability of the recognition process. These statements have been added in line 51-58 on pages 2 to 3.

2. The public URL for the raw data was inaccessible at the time of review.

Response: Thank you for pointing this out. We have updated the dataset again. It can be downloaded normally now.

3. No cross-validation is reported. Please include k-fold cross-validation or a leave-one-session-out evaluation.

Response: Thanks for the reviewer’s kind suggestion. The fitness function of PSO adopts ten-fold cross-validation. The fitness function is defined in lines 355-363 on pages 16 to 17.

4. Line 54: The manuscript claims "real-time recognition" yet provides no latency or computational-resource metrics.

Response: Thank you for pointing this out. We have added an introduction to the algorithm execution environment and a comparison of the average recognition time of different methods, including the method proposed in this paper in lines 292-294 and 377-392 on pages 13 and 17 to 19.

5. Although PSO is used to tune the penalty factor and kernel parameter, key hyper-parameters such as swarm size, maximum iterations, and inertia weights are missing.

Response: Thank you for pointing this out. In the paper, swarm size is 60, maximum iterations is 200, acceleration coefficients are 1.5 and 2.0, inertia factor is 1.0. The negative average recognition accuracy for recognizing porcine abnormal sounds is used as the fitness function, determined through ten-fold cross-validation. These details have been added in lines 353-356 on pages 16 to 17.

6. The dataset is small. Please add a discussion of its limitations.

Response: Thanks for the reviewer’s kind suggestion. SVDD is an one-class classifier. Its basic idea is to construct the smallest sphere which contains all possible training data. Therefore, SVDD does not require a substantial amount of training data. Subsequently, we will analyze the impact of the number of training samples on the model's recognition accuracy. These details have been added in lines 418-419 on page 19.

Thank you very much for your constructive comments and suggestions which would help us both in English and in depth to improve the quality of the paper.

Kind regards,

Sunan Zhang
